# A prospective observational study of the prevalence and risk factors for colonization by antibiotic resistant bacteria in patients at admission to hospital in Singapore

**DOI:** 10.1186/1471-2334-14-298

**Published:** 2014-06-02

**Authors:** Barnaby E Young, David C Lye, Prabha Krishnan, Siew Pang Chan, Yee Sin Leo

**Affiliations:** 1 Institute of Infectious Diseases and Epidemiology, Communicable Diseases Centre, Singapore, Singapore; 2 Department of Medicine, National University of Singapore, Singapore, Singapore; 3 Department of Laboratory Medicine, Tan Tock Seng Hospital, Singapore, Singapore; 4 Department of Mathematics & Statistics, La Trobe University, Melbourne, Australia

**Keywords:** Antibiotics, Colonization, ESBL, Fecal carriage, MRSA

## Abstract

**Background:**

Drug resistant organisms pose an increasing threat to the successful treatment of common infections. Understanding colonization patterns of these bacteria is important for effective antibiotic treatment and infection control guidelines.

**Methods:**

A prospective observational study was performed to determine the prevalence of colonization with extended-spectrum β-lactamase-producing Enterobacteriaceae (ESBL-E), methicillin-resistant *Staphylococcus aureus* (MRSA), and vancomycin-resistant *Enterococcus* (VRE) among patients admitted via the emergency department to a public tertiary hospital in Singapore. Anterior nares, groin, axillary and rectal swabs were collected at admission and cultured using standard bacteriological techniques. Clinical data including healthcare contact within the past 12 months and recent antibiotic use was collected and analyzed using a logistic regression model.

**Results:**

1006 patients were screened. 124 (12.4%) were colonized by ESBL-E, 18 (1.8%) by MRSA while no VRE was detected. Antibiotic use within the past month was the only significant predictor for ESBL-E colonization in the regression model, with an adjusted odds ratio (AOR) of 2.58 (1.04 to 6.42). In participants recently prescribed antibiotics and hospitalized in the previous 3 months, 29.4% were colonized by ESBL-E. This represented 20.2% of the total ESBL-E burden, and ESBL-E was also detected in 6.3% of participants with no healthcare contact. Hospitalization and outpatient hospital visits predicted MRSA colonization in the univariate analysis. Neither was statistically significant in the logistic regression model, with AORs for MRSA colonization following hospitalization in the past 3 and 12 months of 3.81 [95% CI 0.84-17.28] and 3.48 [0.64-18.92] respectively.

**Conclusion:**

A high prevalence of colonization with ESBL-E was evident among patients at admission, even in the absence of recent antibiotic use or contact with healthcare.

## Background

Methicillin-resistant *Staphylococcus aureus* (MRSA) and vancomycin-resistant *Enterococcus* (VRE) emerged in continental Asia following dissemination from Europe and North America [1]. Over the past decade these pathways have reversed, with Asia the probable source of broad-spectrum β-lactamases such as CTX-M-15 and NDM-1 [2,3]. Both are now identified globally.

VRE is relatively uncommonly identified in Asia, while MRSA has become established in Asian hospitals and is emerging in the community [4]. Extended-spectrum β-lactamase-producing Enterobacteriaceae (ESBL-E) have reached an extraordinary prevalence in clinical isolates from both hospitals and the community. Surveillance by the Study for Monitoring Antimicrobial Trends (SMART) in the Asia-Pacific region from 2008–2010 detected ESBL-E in 32.8% of urinary tract infections and 11.2% of intra-abdominal infections (IAI) [5,6]. Almost 20% of ESBL-E were cultured from community-onset IAI. ESBL-E fecal carriage rates among healthy volunteers in Asia have ranged from 6.4% in Japan to an extraordinary 61.7% in rural Thailand [7,8].

As many bacterial infections derive from host flora, colonization by drug resistant organisms is a risk for drug resistant infections [9,10]. An increased mortality with these infections correlates with delayed recognition and inadequate empirical therapy [11,12]. Knowledge of local resistance patterns is therefore important for evidence-based institutional empiric antibiotic treatment and infection control guidelines. We developed a prospective study to determine the prevalence of colonization by ESBL-E, MRSA and VRE in patients admitted to our hospital in Singapore, and to explore some of the associated risk factors.

## Methods

### Participants and setting

We conducted this prospective observational study at the emergency department (ED) of Tan Tock Seng Hospital, Singapore. This is a public tertiary hospital of more than 1500 inpatient beds and a busy ED with more than 11,000 attendances and almost 4000 admissions per month in 2006.

Consecutive patients over 16 years of age planned for hospital admission were approached to participate. The recruitment period was 12-8 pm Monday to Friday from November 2006 to February 2007. Pregnant women were excluded. Target recruitment was 1000, calculated to estimate the colonization rate for MRSA with 95% confidence within 1% of its actual value, based on an expected value of 2% from previous institutional surveillance studies.

Anterior nares, groin, axilla and rectum were swabbed by a research nurse in the ED. If the participant declined or was unsuitable for rectal swabbing, freshly passed stools were collected within 24 hours of admission. Clinical data recorded at admission from the participant or next-of-kin included age, gender, co-morbidities, residence in a care facility, hospitalization or outpatient (hospital and primary care) healthcare contact within the last 12 months and receipt of antibiotics in the last 3 months.

Written informed consent was obtained from all participants or the next-of-kin and the study was approved by the Institutional Ethics Committee (the National Healthcare Group Domain Specific Review Board).

### Bacteria identification and resistance testing

Rectal swabs/stool specimens were inoculated onto two MacConkey agar plates supplemented with 1 mg/L of either cefotaxime or ceftazidime and incubated for 48 hours. Enterobacteriaceae were identified by Microbact 12A (Oxoid, UK) and the presence of ESBL screened with double-disc synergy using cefotaxime 30 μg and ceftazidime 30 μg discs placed 25 mm from an amoxicillin-clavulanate (20 μg-10 μg) disc. Isolates screening positive for ESBL were tested with a multiplex PCR assay for detection of CTX-M clusters, according to published protocols [13].

Nares, groin and axillary swabs were inoculated onto mannitol salt agar supplemented with oxacillin 6 μg/ml and incubated for 48 hours. MRSA was identified by gram stain, tube coagulase test and antimicrobial susceptibility testing.

Rectal swabs/stool specimens were also inoculated onto bile-esculin-azide agar containing 6 mgs/L vancomycin for 48 hours and black colonies identified as enterococci by conventional methods including motility and pigment production. Vancomycin resistance was confirmed using vancomycin minimum concentration (MIC) E-test (AB BioDisk, Sweden) and an in-house *van* gene polymerase chain reaction (PCR).

Disc diffusion anti-microbial susceptibility testing was performed for all isolates in accordance with the 2007 Clinical Laboratory Standard Institute Guidelines [14].

### Data analysis

An initial exploratory analysis was performed with Fisher’s exact test. Logistic regression was used to determine which covariates were significantly associated with the detection of ESBL-E, MRSA or VRE. All identified covariates were entered into the model before employing a backward elimination algorithm. Goodness-of-fit was estimated with the Hosmer Lemeshow (H-L) test. The sensitivity and specificity of individual measures for predicting colonization was estimated using the efficient-score method.

Participants who reported no contact with healthcare, healthcare workers, antibiotics or nursing homes were used as a surrogate for the community prevalence of colonization by ESBL-E, MRSA or VRE.

Statistical analyses were performed using Stata 12.0 (Stata Corporation, U.S.A.). All statistical tests were conducted at a two-sided 5% level of significance.

## Results

1006 out of 1592 patients (63.2%) admitted during the recruitment period consented to participate. Rectal swabs were obtained from 1001 (99.5%); 3 (0.3%) provided stool specimens; 2 (0.2%) did not provide any sample. MRSA screening was performed on all 1006.

Baseline characteristics and findings are summarized in Table 1. 124 (12.4%) were colonized by ESBL-E, 18 (1.8%) by MRSA, and no VRE was detected.

**Table 1:** Sample characteristics and univariate analysis

	**MRSA No (n = 988)**	**Yes (n = 18)**	**p-value**	**ESBL-E No (n = 880)**	**Yes (n = 124)**	**p-value**
**Demographics:**						
Gender						
Male	643 (98.2%)	12 (1.8%)	NS	571 (87.2%)	84 (12.8%)	NS
Female	345 (98.3%)	6 (1.7%)		309 (88.5%)	40 (11.5%)	
Ethnicity						
Chinese	639 (97.6%)	16 (2.4%)	NS	571 (87.2%)	84 (12.8%)	NS
Malay	166 (99.4%)	1 (0.6%)		149 (89.2%)	18 (10.8%)	
Indian	133 (99.2%)	1 (0.8%)		115 (86.5%)	18 (13.5%)	
Others	50 (100.0%)	0 (0.0%)		45 (91.8%)	4 (8.2%)	
Residence in Nursing Home						
No	966 (98.2%)	18 (1.8%)	NS	860 (87.6%)	122 (12.4%)	NS
Yes	22 (100.0%)	0 (0.0%)		20 (90.9%)	2 (9.1%)	
**Comorbid conditions:**						
Diabetes Mellitus						
No	782 (98.7%)	10 (1.3%)	0.035	697 (88.2%)	93 (11.8%)	NS
Yes	206 (96.3%)	8 (3.7%)		183 (85.5%)	31 (11.5%)	
ESRF						
No	977 (98.2%)	18 (1.8%)	NS	869 (87.5%)	124 (12.5%)	NS
Yes	11 (100.0%)	0 (0.0%)		11 (100.0%)	0 (0.0%)	
Liver Cirrhosis						
No	977 (98.2%)	18 (1.8%)	NS	869 (87.5%)	124 (12.5%)	NS
Yes	11 (100.0%)	0 (0.0%)		11 (100.0%)	0 (0.0%)	
Cancer						
No	962 (98.2%)	18 (1.8%)	NS	854 (87.3%)	124 (12.7%)	NS
Yes	26 (100.0%)	0 (100.0%)		26 (100.0%)	0 (0.0%)	
HIV						
No	985 (98.2%)	18 (1.8%)	NS	878 (87.7%)	123 (12.3%)	NS
Yes	3 (100.0%)	0 (0.0%)		2 (66.7%)	1 (33.3%)	
Corticosteroids						
No	944 (98.2%)	17 (1.8%)	NS	845 (88.1%)	114 (11.9%)	NS
Yes	43 (97.7%)	1 (2.3%)		34 (77.3%)	10 (22.7%)	
**Healthcare risk factors:**						
Hospitalization						
Within 3 month	155 (93.4%)	11 (6.6%)	<0.001	131 (78.9%)	35 (21.1%)	<0.001
Within 12 month	297 (95.5%)	14 (4.5%)	<0.001	257 (82.6%)	54 (17.4%)	0.002
Oral antibiotics						
Within 1 month	279 (97.2%)	8 (2.8%)	NS	227 (79.1%)	60 (20.9%)	<0.001
Within 3 month	343 (97.4%)	9 (2.6%)	NS	286 (81.3%)	66 (18.7%)	<0.001
Outpatient hospital visit						
Within 3 month	402 (96.9%)	13 (3.1%)	0.013	347 (83.8%)	67 (16.2%)	0.002
Within 12 month	536 (97.3%)	15 (2.7%)	0.016	470 (85.4%)	80 (14.6%)	0.021
Primary care visit						
Within 3 month	657 (97.6%)	16 (2.4%)	0.046	584 (87.0%)	87 (13.0%)	NS
Within 12 month	729 (97.9%)	16 (2.1%)	NS	652 (87.8%)	91 (12.2%)	NS
HCW contact						
No	853 (98.3%)	15 (1.7%)	NS	759 (87.5%)	108 (12.5%)	NS
Yes	135 (97.8%)	3 (2.2%)		121 (88.3%)	16 (11.7%)	

ESRF: End-stage Renal Failure; HIV: Human Immunodeficiency Virus; HCW: Healthcare Worker.

### Univariate analysis and logistic regression

Inpatient or outpatient hospital care and recent antibiotic prescription were significant risk factors for ESBL-E colonization in the univariate analysis (Table 1). In the logit model only antibiotic use within 1 month prior to admission significantly predicted ESBL-E colonization, with an AOR of 2.58 [1.04 to 6.42] (Table 2).

**Table 2:** Multiple logistic regression analyses of MRSA and ESBL-E colonization risk factors

Featured covariates*	**MRSA AOR**	**95% C.I.**	**ESBL-E AOR**	**95% C.I.**
Hospitalization				
Within 3 month	3.81	0.84 to 17.28	1.38	0.71 to 2.69
Within 12 month	3.48	0.64 to 18.92	1.35	0.73 to 2.48
Use of oral antibiotics				
Within 1 month	1.57	0.17 to 14.43	2.58**	1.04 to 6.42
Within 3 month	0.67	0.08 to 6.78	0.94	0.38 to 2.36
Outpatient visit				
Within 3 month	0.97	0.17 to 5.64	1.36	0.69 to 2.69
Within 12 month	1.43	0.18 to 11.13	0.89	0.43 to 1.80
Primary care visit				
Within 3 month	Not estimated	Not estimated	2.08	0.72 to 6.01
Within 12 month	Not estimated	Not estimated	0.41	0.14 to 1.24
Contact with Healthcare Worker	1.30	0.32 to 5.33	0.86	0.47 to 1.57

*The models are adjusted for demographics and co-morbid conditions.

**Statistically significant at 5%.

For MRSA colonization, inpatient or outpatient hospital care within the previous 3 or 12 months were significantly associated in the univariate analysis (Table 1). Neither factor was statistically significant in the logit model. Adjusted odds ratios (AOR) for MRSA colonization following hospitalization in the past 3 and 12 months were 3.81 [95% CI 0.84-17.28] and 3.48 [0.64-18.92] respectively (Table 2).

Both logit models provided a satisfactory fit to the data (H-L test: ESBL-E p-value = 0.52, MRSA p-value = 0.87).

### Community colonization

95 participants reported no contact with healthcare, healthcare workers, antibiotics or nursing homes; 6 (6.3%) were colonized by ESBL-E, 0 by MRSA and 0 by VRE.

The importance of recent antibiotic use for ESBL-E colonization was evident from a comparison of colonization rates in participants stratified by attendance at different levels of the healthcare system in the previous three months, and antibiotic use in the previous month (Table 3). Baseline colonization prevalence in each subgroup was not significantly different from the putative community prevalence of 6.3% (Fisher’s exact test), however prevalence was significantly higher than baseline if antibiotics were administered in the previous month. In participants who had been recently admitted to hospital and received antibiotics, 29.4% were colonized by ESBL-E.

**Table 3:** ESBL-E fecal colonization by healthcare contact in the past three months and antibiotic use in the previous month

**Sub-group**	**Number of participants**		**Prevalence**	**Odds ratio (95% C.I.)**	**p-value**
	**ESBL-E colonized**	**Not colonized**			
No healthcare contact or antibiotics	6	95	6.3%		
Primary care, no antibiotics	36	452	8.0%	2.90* (1.84 to 4.57)	<0.0001
Primary care and antibiotics	51	221	23.1%		
Outpatient, no antibiotics	24	245	9.8%	1.78* (1.05 to 3.02)	0.033
Outpatient and antibiotics	43	247	17.4%		
Hospitalization, no antibiotics	10	81	12.3%	2.38* (1.08 to 5.02)	0.032
Hospitalization and antibiotics	25	85	29.4%		

*Statistically significant at 5%.

### Predicting colonization

Oral antibiotics in the past month predicted ESBL-E fecal carriage with a sensitivity of 48.4% [95% CI 39.4–57.5%] and specificity of 74.2% [71.2–77.0%]. Specificity could be improved by filtering for additional hospitalization in the past 3 months, but with a significant loss of sensitivity: this was 22.1% [15.3-28.2%] sensitive and 89.4% [87.0-91.3%] specific. Specificity was improved further by combining oral antibiotics in the past month and hospitalization within the past 3 months. This predicted ESBL-E fecal carriage with 20.2% [13.7–28.5%] sensitivity and 93.2% [91.3-94.7%] specificity.

Hospitalization within the last 3 months was 61.1% [95% CI 36.1-81.7%] sensitive and 84% [81.9-86.5%] specific for MRSA colonization. Sensitivity could be improved to 77.8% [51.9-92.6%], but with a specificity of 69% [70.0-72.8%] using hospitalization in the past 12 months to predict MRSA colonization.

### Extended spectrum β-lactamases

133 Enterobacteriaceae isolates from 124 participants tested positive for ESBL production by double disc synergy. 71% of these were *E.coli* and 19% *K.pneumoniae.* 99 (74.4%) were PCR positive for at least one CTX-M enzyme, predominantly cluster 1 (64 isolates, 65%) or 9 (32 isolates, 24%). 3 isolates produced both cluster 1 and 9 CTX-M.

## Discussion

Ceftriaxone resistance among clinical isolates of *E.coli* and *K.pneumoniae* from public hospitals in Singapore has reached 20-30% [15]. This incidence is rising and correlates with increasing prescription of broad spectrum antibiotics. We identified a similar high burden of ESBL-E colonization from rectal swab cultures. ESBL-E was detected in 29.4% of subjects who had been recently hospitalized and were recipients of antibiotics. 6.3% of subjects without healthcare contact or antibiotics were detected as ESBL-E colonized. The source of these organisms is of major concern.

In ESBL-E high prevalence countries such as Singapore, nosocomial transmission may not be the most important acquisition route. A study in a Swiss hospital with low ESBL-E prevalence observed infrequent transmission events even without contact isolation [16]. Modeling ESBL-E colonization in a French pediatric unit described the success of contact isolation in preventing nosocomial transmission, but its overall ineffectiveness due to the high incidence of sporadic cases from the community [17]. A similar finding from a study of ESBL-E transmission dynamics in Switzerland, detected more inpatient ESBL-E fecal carriage acquired from the community than in hospital [18]. Transmission between household members was also more frequent than between patients, though it was not clear if it was acquired from a common source – presumed to be food – or person-to-person.

The importance of recent antibiotic use in this study suggests that host susceptibility to colonization is a critical factor. Acquisition of ESBL-E from community or nosocomial sources may be facilitated by disrupting the host gut microbiome and providing a selective advantage for antibiotic-resistant organisms. Given the high rate of colonization in patients with no identified risk factors, an alternative hypothesis is that antibiotic use selects for ESBL-E from host indigenous gut flora, increasing the colonization burden. The sensitivity of rectal swabs for detection of ESBL-E has not been widely investigated, but was reported to correlate with density of colonization [19]. Antibiotics also generate *de novo* ESBL-E, by promoting DNA recombination events between gut bacteria [20].

Travel to Asia has been identified as a risk factor for ESBL-E fecal carriage in European and Australian prospective cohorts [21,22]. In both these studies, acquisition was associated with traveler’s diarrhea, but not dependent on antibiotic use or healthcare contact. Curiously this is despite the isolation of ESBL-E from more than 90% of retail chicken meat in separate European studies [23,24]. This disconnect could be due to environmental or diet differences facilitating ESBL-E acquisition in Asia, or microbiome changes as a result of travel itself increasing susceptibility to colonization by antibiotic-resistant bacteria. CTX-M production by *E.coli* carried on chicken, pigs and cattle in China has been reported, and a systematic study of agricultural, food and clinical ESBL-E isolates in Asia would be interesting [25].

MRSA is a common healthcare-associated pathogen in Singapore. A colonization prevalence of 41% has been reported from nursing homes residents admitted to a public acute care hospital, and 6% among all medical and surgical admissions [26]. Community-acquired MRSA strains are infrequently recognized as a cause of infections in Singapore, and its contribution to community MRSA colonization is not known [27].

Transmission of MRSA from colonized hands and environmental surfaces to new hosts is well established [28,29]. The high AOR in the logit model between hospitalization and MRSA colonization is consistent with this as a clinically significant factor. Antibiotic use has also been associated with colonization and infection by MRSA. Failure to identify this here may be due to the small number of cases [30,31]. Failure to isolate VRE was not unexpected, as the prevalence in Singapore is low outside of nosocomial outbreaks [32].

This study has a number of limitations. Accurate clinical data was dependent on recall by study participants or next-of-kin and was not independently verified by medical records. Data was not collected on antibiotic type and duration or frequency of prior contact with healthcare institutions as this was judged unreliable from patient recall. Our ability to analyze risk factors for colonization by MRSA and VRE was limited by the study design, due to the low detected prevalence. A case control study would help elaborate these.

Acquisition rates in hospital were not investigated, and this could have provided additional evidence to refine our conclusions. More accurate methods for detecting MRSA, ESBL-E and VRE colonization are now available: for example PCR-based detection methods may be more accurate and sensitive [33]. We were also unable to perform ESBL genotyping or bacterial strain typing, to determine if CTX-M-15 was the major enzyme in clusters 1 and 9, or if ESBL-E strains differed between apparent community- and hospital-acquired isolates. The prevalence of resistance mechanisms such as carbapenemases which have become of increasing concern was not assessed.

## Conclusions

ESBL-E colonization was detected with high prevalence in subjects who were recently hospitalized and received antibiotics. Identifying subjects who were colonized by ESBL-E accurately was difficult using just the clinical data collected. Not least this was due to the significant proportion of ESBL-E associated with limited healthcare contact. Identifying the source of these organisms and the relative contributions of community and nosocomial transmission may be important for understanding how to control their spread.

## Abbreviations

AOR: Adjusted odds ratio; CI: Confidence interval; ED: Emergency department; ESBL-E: Extended-Spectrum β-lactamase-producing Enterobacteriaceae (ESBL-E); ESRF: End-stage renal failure; HCW: Healthcare worker; HIV: Human immunodeficiency virus; H-L: Hosmer Lemeshow; IAI: Intra-abdominal infection; OR: Odds ratio; PCR: Polymerase chain reaction; MRSA: Methicillin-resistant *Staphylococcus aureus*; VRE: Vancomycin-resistant *Enterococcus*.

## Competing interests

The authors declare that they have no competing interests.

## Authors’ contributions

BY collected and analyzed data, and drafted the manuscript; PK conceived and designed the study, and performed culture and susceptibility testing; SC analyzed data and helped draft the manuscript; DL conceived and designed the study, collected and analyzed data and helped draft the manuscript; LY conceived and designed the study. All authors read and approved the final manuscript.

## Pre-publication history

The pre-publication history for this paper can be accessed here:

http://www.biomedcentral.com/1471-2334/14/298/prepub
